# A Potentiometric Formaldehyde Biosensor Based on Immobilization of Alcohol Oxidase on Acryloxysuccinimide-modified Acrylic Microspheres

**DOI:** 10.3390/s101109963

**Published:** 2010-11-05

**Authors:** Yew Pei Ling, Lee Yook Heng

**Affiliations:** School of Chemical Sciences and Food Technology, Faculty of Science and Technology, Universiti Kebangsaan Malaysia (UKM), 43600 Bangi, Selangor Darul Ehsan, Malaysia; E-Mail: peiling840917@yahoo.com

**Keywords:** polymer spheres, *n*-butyl acrylate, *N*-acryloxysuccinimide, potentiometric biosensor, photopolymerization

## Abstract

A new alcohol oxidase (AOX) enzyme-based formaldehyde biosensor based on acrylic microspheres has been developed. Hydrophobic poly(*n*-butyl acrylate-*N*-acryloxy-succinimide) [poly(nBA-NAS)] microspheres, an enzyme immobilization matrix, was synthesized using photopolymerization in an emulsion form. AOX-poly(nBA-NAS) microspheres were deposited on a pH transducer made from a layer of photocured and self-plasticized polyacrylate membrane with an entrapped pH ionophore coated on a Ag/AgCl screen printed electrode (SPE). Oxidation of formaldehyde by the immobilized AOX resulted in the production of protons, which can be determined via the pH transducer. Effects of buffer concentrations, pH and different amount of immobilization matrix towards the biosensor’s analytical performance were investigated. The formaldehyde biosensor exhibited a dynamic linear response range to formaldehyde from 0.3–316.2 mM and a sensitivity of 59.41 ± 0.66 mV/decade (R^2^ = 0.9776, n = 3). The lower detection limit of the biosensor was 0.3 mM, while reproducibility and repeatability were 3.16% RSD (relative standard deviation) and 1.11% RSD, respectively (n = 3). The use of acrylic microspheres in the potentiometric formaldehyde biosensor improved the biosensor’s performance in terms of response time, linear response range and long term stability when compared with thick film immobilization methods.

## 1. Introduction

Formaldehyde (H_2_CO) is the simplest aldehyde compound. It is commonly used as a disinfectant and biocide [1] and as a preservative in the food industry [2]. Formaldehyde is also naturally present in living organisms, fruits, vegetables and biological compounds [3,4]. Relatively high concentration of formaldehyde can be found in seafood and crustaceans, due to the enzymatic dissociation of trimethylamine-oxide (TMAO) [5].

TMAO is often found in fish as a natural compound that exists in their muscles [6]. The formation of formaldehyde in fishery products is very dependent on the environment during the storage process and some fish species can produce up to 200 mg kg^−1^ of formaldehyde even under frozen storage conditions. A high content of accumulated formaldehyde in food poses a threat to human health [5], as formaldehyde is toxic, allergenic and carcinogenic and can cause symptoms like headaches, burning sensation in the throat and difficulty in breathing [1] and it has been declared a potential carcinogen and mutagen [2].

Consequently, formaldehyde monitoring in the environment and food samples is crucial and various analytical methods for determining trace amount of formaldehyde have been developed [7]. Spectrophotometry [2,8–12], fluorimetry [13–16] and colorimetry [6] are popular techniques based on color changes upon reaction with formaldehyde but they are not favorable for *in-situ* applications because these methods require preparation of various color forming reagents and typically have slow response times (6 min to a few hours). Other standard methods such as high-performance liquid chromatography (HPLC) [2,17–23] and gas chromatography (GC) [24,25] are well known for formaldehyde detection where 2,4-dinitrophenylhydrazine is commonly used as a derivitization agent for such techniques. Both chromatographic and colorimetric methods suffer to certain extent interference from other carbonyl substances, especially acetaldehyde and acetone, not to mention the fact the detection techniques involve tedious derivative procedures and the use of expensive and complicated instrumentation [26].

On the other hand, biosensors show potential for complementing both laboratory-based and field analytical methods for food monitoring. Enzyme immobilization is one of the most important facets in biocatalysis-based biosensors research. When an enzyme is immobilized in a polymer matrix, access of analyte or products via diffusion must occur, but the enzyme should be retained. Covalent immobilization via polymer matrices benefits from the loss prevention of enzymes and sometimes better enzyme stabilization [27]. Application of nano/micro-sized matrix materials for covalent enzyme attachment is becoming popular because of their large surface area, which improves the enzyme binding capacity and increases the mass transfer kinetics when the enzymatic reaction occurs at the surface of nano/micro-sized matrix materials, compared with in the polymer film matrix [28].

Most reported sensors based on polymer microspheres were ion sensors [29–36]. Polymeric microspheres and nanospheres have been used for enzyme immobilization but their application to biosensor is still rather unexplored. Bayramoğlu *et al.* [37] have used poly(2-hydroxyethyl methacrylate-*co*-*N*-methacryloly-l-histidinemethylester) microspheres containing l-histidine groups chelated with Ni(II) ions for urease immobilization and found that there was an increase in enzyme stability and improvement in the range of optimum enzyme operational temperature. Brahim *et al.* [38] immobilized glucose oxidase into crosslinked poly(hydroxyethyl methacrylate-*co*-dimethylaminoethyl methacrylate) hydrogel microspheres and confirmed that the hydrogel microsphere matrix presented no significant diffusional barrier to enzyme-substrate reaction. Polymeric nanospheres from thiol-functionalized poly(divinylbenzene-*co*-acrylic acid) have been used for self-assembly of gold nanoparticles and horseradish peroxidase immobilization to fabricate amperometric biosensors for hydrogen peroxide detection. The resulting biosensors showed a large improvement in linear range, exhibited high sensitivity, good reproducibility, and long-term stability [39–41].

In view of the advantages provided by the use of polymeric microspheres for enzyme immobilization, the aim of this study was to develop a novel enzyme-based formaldehyde biosensor where the enzyme alcohol oxidase (AOX) is covalently immobilized on a new type of acrylic microspheres. These acrylic microspheres were hydrophobic in character with a surface modified acryloxysuccinimide functionality (poly-nBA-NAS) for the immobilization of the enzyme. They were synthesized via photopolymerization. As the microspheres are hydrophobic, the AOX immobilization will be confined to the surface of the spheres, thus allowing the enzymatic reaction of AOX and formaldehyde to occur at the surface. With a large surface area of the microsphere to be use as a potetiometric biosensor membrane and favorable surface diffusion conditions, the analytical performance of the formaldehyde biosensor can be improved.

## 2. Experimental Section

### 2.1. Materials

2,2-Dimethoxy-2-phenylacetophenone (DMPP), sodium tetrakis [3,5-bis (trifluro-triethyl) phenyl] borate (NaTFPB), hydrogen ionophore I (tridodecylamine), sodium dihydrogen phosphate (NaH_2_PO_4_) were obtained from Fluka. Sodium hydroxide (NaOH), sodium dodecyl sulphate (SDS), acetic acid, acetyl acetone from Systerm. In addition, 2-hydroxyethyl methacrylate (HEMA), poly(HEMA) commercial, 2-hexanediol diacrylate (HDDA), alcohol oxidase enzyme (AOX) from *Hansenula polymorpha*, bovine serum albumin (BSA), Bradford reagent, all were from Sigma Aldrich. N-acryloxysuccinimide (NAS) and tris(hydroxymethyl) aminomethane (Tris-HCl) were purchased from Acros Organics and Duchefa Biochemie, respectively. Formaldehyde solution was obtained from BDH, *n*-butyl acrylate (nBA) from Merck, hydrochloride acid 37 % (HCl) from Riedel-de Haen, di-sodium hydrogen phosphate (Na_2_HPO_4_) from Hamburg Chemical, ammonium acetate from Scharlau while both Bactor agar and 1,4-dioxane were from Ajax Chemicals. All chemicals were of analytical grade and used without further purification. Standard buffer solutions were prepared with deionized water.

### 2.2. Synthesis of Poly(nBA-NAS) Microspheres

Poly(nBA-NAS) microspheres were prepared via photopolymerization in the form of an emulsion. A mixture of 4 mL of nBA monomers, 0.09 g DMPP, 400 μL HDDA, 0.1 g SDS, 10 mg NAS and 10 mL deionized water was prepared in a sample bottle. The resulting emulsion turned milky white after sonication for 5 min. The milky solution was then photocured for 300 s under continuous purging with nitrogen gas in an ultraviolet exposure unit (R.S. Ltd.) of 15 Watt light intensity at a wavelength of 350 nm. Poly(nBA-NAS) microspheres were isolated by centrifugation (4,000 rpm, KUBOTA) for 8 min and finally washed a few times with 0.01 M sodium phosphate buffer solution (pH 8.0). Clean poly(nBA-NAS) microspheres were dried at room temperature and kept at 4 °C when not in use.

### 2.3. Determination of Size and Distribution of Poly(nBA-NAS) Microspheres

The shape and size of poly(nBA-NAS) microspheres were investigated using a scanning electron microscopy (SEM, LEO 1450VP) at a acceleration voltage of 20 kV. Dry poly(nBA-NAS) microspheres were placed on a piece of glass slide and then deposited with a thin layer of gold to reduce the charge effect from primary electron beam, which may cause scanning faults [31]. Size and distribution of poly(nBA-NAS) microspheres were determined based on a random selection of 264 microspheres from a scanning electron micrograph.

### 2.4. Optimization of Enzyme Binding

Bradford protein assay was conducted to determine enzyme binding so to ascertain the optimum amount of NAS required in the preparation of poly(nBA-NAS) microspheres [42]. For this purpose, 1.4 g of poly(nBA-NAS) microspheres for each NAS content (NAS = 5; 10; 15; 20; 25 mg) were placed on a screen-printed electrode (SPE) and dried at 4 °C. After 24 hr, 2 μL AOX solution (0.05 mg μL^−1^) was dropped onto the surface of poly(nBA-NAS) microspheres deposited on SPE and left at 4 °C for 24 hr. Finally the SPE with immobilized enzyme on the spheres was immersed in 3 mL of 0.05 M phosphate buffer solution at pH 8 for 30 min. To determine the amount of enzyme present in the wash solution of the microspheres, a mixture of 100 μL phosphate buffer washing, 100 μL NaOH and 800 μL Bradford reagent was mixed and incubated for 6 min. The absorbance (at 595 nm) of the mixture was measured using a spectrophotometer (Cary 50). For calibration of the Bradford microassay, a series of standard BSA was prepared (0; 10; 20; 30; 40 and 50 μg mL^−1^) in 0.05 M sodium phosphate buffer at pH 8.

### 2.5. Fabrication of Formaldehyde Biosensor

Before the fabrication of the potentiometric formaldehyde biosensor, first the H^+^ ion sensor transducer was prepared. Procedures for the fabrication of H^+^ ion sensor were as reported elsewhere [43] but with minor modifications in this experiment. A mixture of HEMA monomers and 1.6 wt% of DMPP was drop-coated onto a Ag/AgCl SPE and photocured for 180 s under nitrogen gas atmosphere. The poly(HEMA) film formed was then hydrated with 0.1 mM Tris-HCl buffer (pH 7.0) for 15 min. The H^+^ ion-selective and plasticizer-free poly(nBA) membrane, which contained 0.1 wt% nBA, 1 wt% HDDA and DMPP, 1.9 wt% hydrogen ionophore I and 0.8 wt% of NaTFPB was prepared on the poly(HEMA) film by photocuring for 180 s under nitrogen gas purging.

The H^+^ ion sensor response was evaluated before it was used as a transducer for the biosensor. Using a double junction Ag/AgCl reference electrode filled with 0.1 M Tris-HCl (pH 7.0) saturated with AgCl and 0.1 M lithium acetate as gel bridge electrolyte, the stable potential differences (EMF, mV) between the H^+^ ion sensor and the reference electrode were measured for a series of 0.1 mM Tris-HCl solutions with pH range of 2–12. The pH of these Tris-HCl solutions were measured with an Ecomet pH meter before use and the pH was adjusted with HCl (0.1 M) and NaOH (0.1 M). The EMF values obtained were then plotted against the logarithmic activities based on the Nernst equation [43–45].

**Figure 1.**
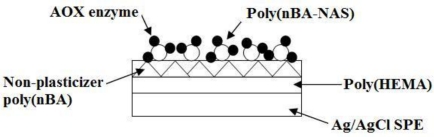
Potentiometric formaldehyde biosensor based on AOX-poly(nBA-NAS) deposited on membranes on a Ag/AgCl SPE.

H^+^ ion sensors with good or close to Nernstian response was then used for the fabrication of formaldehyde biosensor based on immobilized AOX-poly(nBA-NAS) microspheres. The AOX solution was prepared by dissolving 2.5 mg AOX (13 unit mg^−1^) in 50 μL 0.05 M sodium phosphate buffer (pH 8.0) and stored at 0 °C before use. The immobilization procedure involved addition of 50 μL AOX solution (0.05 mg μL^−1^) and 700 μL of 0.05 M sodium phosphate (pH 8.0) to 100 mg dried poly(nBA-NAS) microspheres. The enzyme solution and poly(nBA-NAS) microspheres were allow to react at 4 °C over 24 h. After the reaction period, the resulting AOX-poly(nBA-NAS) microspheres were washed several times with 0.01 M phosphate buffer at pH 8 and later centrifuged at 4000 rpm for 5 min before the spheres were collected. An amount 0.5 mg of dry AOX-poly(nBA-NAS) microspheres was weighed and deposited on top of the H^+^ ion transducer (Figure 1).

For comparison with a formaldehyde biosensor fabricated from poly(HEMA) film as an enzyme immobilization matrix, a mixture of 1.2 mg AOX (13 unit mg^−1^) and 50 μL poly(HEMA) polymer solution (prepared in 1,4-dioxane:deionized water = 1:4) was prepared and left to dissolve completely at 4 °C for 24 h. Finally 2 μL of the AOX and poly(HEMA) mixture was coated onto the H^+^ ion transducer and left to dry at 4 °C for 24 h.

### 2.6. Evaluation of the Response of the Formaldehyde Biosensor

By connecting to a double-junction Ag/AgCl electrode as mentioned above, the response of a formaldehyde biosensor in formaldehyde standard solutions from 0.1–316.2 mM in 10 mM Tris-HCl buffer (pH 6.5) was evaluated. The response of the biosensor (mV) was then plotted against the logarithmic concentrations of formaldehyde. Both the formaldehyde biosensor from poly(HEMA) film and blank electrode without immobilized AOX were tested similarly. The sensitivity, linear range, detection limit, response time, repeatability, reproducibility and long-term stability were assessed.

### 2.7. Effect of Buffer pH and Concentrations on Biosensor Response

The effects of concentration and pH of Tris buffer on the response of the formaldehyde biosensors and blank electrode were investigated in 0.1–316.2 mM formaldehyde solutions. Buffer concentrations (Tris-HCl, pH 6.5) used were varied from 0.1–100 mM. For pH effect, the pH was varied from 5.8 to 7.5 using 10 mM Tris-HCl.

### 2.8. Effect of AOX-Poly(nBA-NAS) Microspheres Loading on Response

To determine the effect of the amount of AOX-microspheres on the biosensor response, biosensors deposited with different amounts of AOX-poly(nBA-NAS) from 0.1–0.6 mg were fabricated and the response of each biosensor was evaluated.

### 2.9. Response Time and Stability Studies of Formaldehyde Biosensor

The response time of the formaldehyde biosensor was investigated with a series of formaldehyde solutions (0.1–316.2 mM) prepared in 10 mM Tris-HCl (pH 6.5). The response was recorded at every 5 s until a stable value was obtained. The response time was taken when the signal was stable with less than 5% fluctuation. Whilst long term stability of biosensor was determined by measuring the biosensor sensitivity over a certain period of time until a decline in response was observed. The formaldehyde biosensor was kept at 4 °C throughout the study. For operational stability evaluation, the response of the biosensor was measured continuously until a response decline was obtained.

### 2.10. Interference and Recovery Studies of Formaldehyde Biosensor

Potential interfering substances towards the formaldehyde biosensor response such as acetaldehyde, methanol, ethanol and glucose were measured separately in the concentration range of 0.1–316.2 mM in 10 mM Tris-HCl (pH 6.5). For the determination of formaldehyde in real samples, 1 g of shrimp sample was meshed and mixed with 5 mL deionized water followed by centrifugation at 4,000 rpm for 30 min to obtain the liquid portion. This liquid was then diluted with Tris-HCl buffer to 10 mM and the pH was adjusted to 6.5. A known concentration of formaldehyde was then added before further analysis using the formaldehyde biosensor. The sample liquid was also analysed using the Nash spectrophotometric procedure. The Nash reagent was prepared by mixing 3 g of ammonium acetate, 60 μL acetic acid and 40 μL acetyl acetone and diluted to 20 mL with deionized water. The reagent was kept in dark and at 4 °C when not in use. For the Nash method, a mixture of 100 μL sample solution containing formaldehyde, 900 μL 10 mM Tris-HCl (pH 6.5) and 10 μL Nash reagent was kept at 60 °C for 15 min before the absorption at wavelength 412 nm was recorded. In the presence of formaldehyde, the solution turned yellow and the concentration of formaldehyde can be obtained from the calibration curve of the Nash method.

## 3. Results and Discussion

### 3.1. The Size Distribution of Poly(nBA-NAS) Microspheres

**Figure 2.**
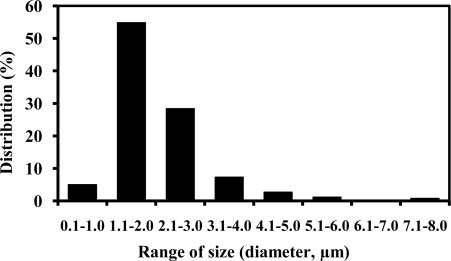
The size distribution pattern of poly(nBA-NAS) microspheres synthesized via photopolymerization method.

In the synthesis of the acrylic microspheres, the water insoluble *n*-butyl acrylate (nBA) monomer was used to prepare high solid content latex and to minimize its coagulation [46]. NAS monomer was chosen for its reactive succinimide groups for the binding to amino-bearing AOX [47]. The long chain alkyl sulphate surfactant, which is amphiphilic was used to stabilize the emulsion system and prevented the monomers from forming larger droplets and allowed small droplets to remain stable during the emulsion phase [28,48]. Photopolymerization caused the droplets of monomers to form poly(nBA-NAS) microspheres at room temperature and this method of polymerization can be terminated simply by removing the light source [49,50]. The poly(nBA-NAS) microspheres synthesized via photopolymerization demonstrated a narrow size distribution, *i.e.*, 0.8–3.0 μm diameter spheres (87.12%) (Figure 2). Uniform size distribution of the microspheres will ensure consistent enzyme binding capacity [28] because of similar surface area of each microsphere.

### 3.2. The Amount of Immobilized AOX Enzyme on Microspheres

**Figure 3.**
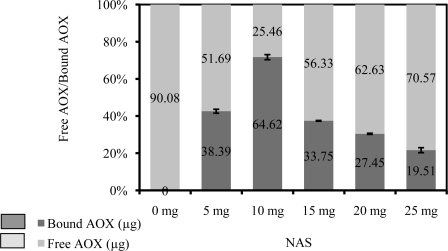
The amount of bound AOX determined according to the amount of NAS used for microspheres synthesis.

From the Bradford microassay, the amount of immobilized enzyme on the poly(nBA-NAS) microspheres with varying composition of NAS was determined. Figure 3 shows that the amount of AOX immobilized on poly(nBA-NAS) microspheres increases when the amount of NAS monomers increases from 5 mg to 10 mg in a nBA-NAS monomers mixture, but it decreases when the NAS monomers used exceeded 10 mg. The high concentration of NAS used can cause reaction of NAS with each other to produce a dimer via Michael addition. This self-reaction occurs between the nucleophilic enolate (succinimide group) and conjugated ketone at the electrophilic alkene where both of these groups located at the opposite end of NAS monomer. This causes the availability of free succinimide groups for coupling with AOX to decline and reduces the total active immobilization sites, thus reducing the binding capacity of the microspheres [28,51,52].

### 3.3. Evaluation of Formaldehyde Biosensor Performance

The potentiometric formaldehyde biosensor is based on a H^+^ ion transducer that deposited with a layer of AOX-poly(nBA-NAS) microspheres. AOX oxidizes formaldehyde to formic acid (HCOOH) and hydrogen peroxide (H_2_O_2_). Formic acid is then dissociated to H^+^ ion:

(1) $${\text{CH}}_{2}O+O_{2}+H_{2}O\to {\text{HCOO}}^{-}+H^{+}+H_{2}O_{2}$$

**Figure 4.**
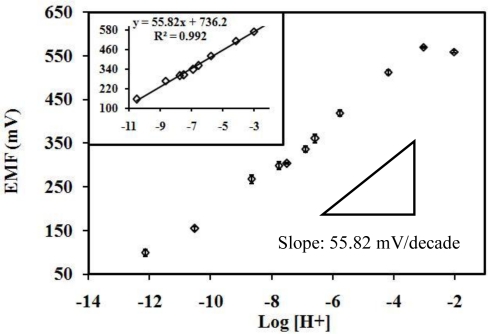
The response of a SPE based H^+^ ion sensor to hydrogen ion concentrations (in 0.1 mM Tris-HCl buffer).

The H^+^ ion selective plasticizer-free nBA membrane detects H^+^ ion from the AOX-poly(nBA-NAS) microspheres. Thus, determination of formaldehyde can be performed by potentiometry via measuring the proton produced from the enzymatic reaction [45] of AOX. Figure 4 shows the H^+^ ion transducer response to pH changes with a slope 55.82 ± 2.9318 mV/decade (R^2^ = 0.9907), which is close to the Nernstian value.

The working linear range of the pH sensor was from pH 3 to 11. Hence this transducer is suitable for the formaldehyde biosensor designed.

#### 3.3.1. Effects of Buffer on Biosensor Response

The formaldehyde solutions in deionized water were acidic, with changes of 0.31 pH unit/decade over the range of 0.1–316.2 mM, as monitored by a pH meter. The pH changes was even greater when formaldehyde solutions were prepared with Tris-HCl buffer. For the formaldehyde concentrations prepared in 0.1 mM and 10 mM Tris-HCl buffers, 0.77 pH unit/decade and 1.00 pH unit/decade change were observed, respectively. The effect of Tris-HCl buffer on the pH pf the formaldehyde solution can be explained by the free H^+^ ion produced when a stronger acid (hydroxymethylamine derivative) formed from the reaction between formaldehyde and the amino group of Tris-HCl [3]:

(2) $${{(\text{HOCH}}_{2})}_{3}{\text{CNH}}_{3}{}^{+}+{\text{CH}}_{2}O={{(\text{HOCH}}_{2})}_{3}{\text{CNHCH}}_{2}\text{OH}+H^{+}$$

This reaction occurs for primary and secondary amines in the presence of aldehydes. Acidification during the reaction can cause the pH to decrease and H^+^ ions released also decrease the buffer capacity [3]. As a result, the response of the formaldehyde biosensor increased when Tris-HCl concentration increased due to the acidification reaction (Table 1).

As shown in Table 2, sensor without AOX enzyme (blank-nano/microspheres-SPE) also produced response because of the acidification reaction. However, with the presence of AOX, the sensor demostrated higher response compared with sensor without AOX. The enzyme AOX catalyses the reaction of formaldehyde to produce H^+^ ion, which increases the proton concentration further to give larger response.

The AOX enzyme has been chosen for the construction of formaldehyde biosensor because of its catalytic activity does not require any external cofactor, it is independent of pH changes over the range of 6 to 10 (optimum pH 7.5–8.0) and its stability at higher temperatures of up to 50 °C [3,52,53]. With immobilization of AOX on to poly(nBA-NAS) microspheres, the enzyme is further protected from direct effect from extreme changes in temperature and pH and hence this increases the stability of AOX by providing an enzyme-friendly microenvironment [54].

The pH of a buffer has effect on the response of the formaldehyde biosensor. From Table 3, the sensitivity increased when pH of Tris-HCl buffer increased. At pH approaching 7, *i.e.*, the optimum pH for AOX under normal conditions, the response became super-Nernstian and a decrease in the linear response range was observed. This is attributed to the higher enzymatic working rate at pH 7 and the acidification effect. Under such conditions, the buffer capacity of Tris-HCl no longer sufficient to control the pH, thus larger fluctuation in response was recorded. At pH below 6.5, enzyme deactivation caused by the acidification effect is less obvious and the linear response range towards formaldehyde is larger and demonstrates better sensor reproducibility. Therefore, buffer at pH 6.5 is more suitable for formaldehyde biosensor operation.

#### 3.3.2. Effect of the Amount of Microspheres on Biosensor Response

The amount of AOX-poly(nBA-NAS) microspheres used in the fabrication of the biosensor affected the biosensor response slightly where the sensitivity was varied by 10 mV/decade over a six fold increase in the enzyme immobilized microspheres (Table 4). But the increase in sensitivity for 0.1 and 0.6 mg of microspheres is statistically significant. Thus the amount of spheres used in the biosensor fabrication affected the total surface area for enzymatic reaction and hence this can affect the performance of the biosensor [39,40].

#### 3.3.3. Optimized Biosensor Performance

**Figure 5.**
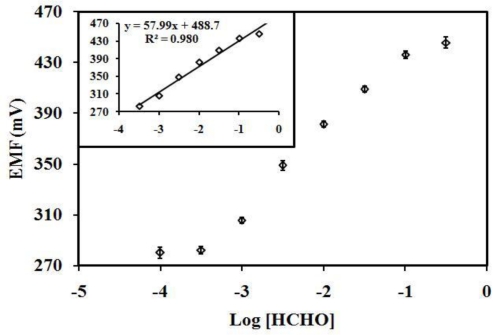
The response of a AOX-poly(nBA-NAS) microspheres based biosensor to formaldehyde in 10 mM Tris-HCl buffer, pH 6.5.

Using optimized values of buffer concentration, pH and the amount of microspheres, the average sensitivity value of the formaldehyde biosensor based on microsphere is 59.41 ± 0.66 mV/decade (R^2^ = 0.9776) in the linear concentration range of 0.3–316.2 mM of formaldehyde. An example of the response curve of the biosensor to formaldehyde is shown in Figure 5.

**Figure 6.**
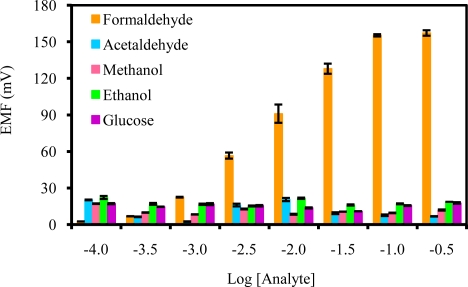
Effects of interfering substances on the response of biosensor based on AOX-poly(nBA-NAS) microspheres in 10 mM Tris-HCl buffer (pH 6.5) as background solution.

The selectivity of the biosensor towards formaldehyde in the presence of some common interfering agents such as acetaldehyde, primary alcohols (methanol and ethanol) and glucose was evaluated. From Figure 6, it is clear that all the potential interfering substances studied do not demonstrate strong interference towards the response of formaldehyde over the concentration range of 3.2–316.2 mM. The enzyme AOX has higher affinity towards methanol when compared with formaldehyde. But when used in potentiometric biosensor, AOX often demonstrated selectivity to formaldehyde instead of methanol and this has been discussed earlier by other researchers [3,55].

Deficiency of oxygen is the main reason for a potentiometric biosensor not responding to methanol. This is because reaction of AOX with methanol requires twice the amount of oxygen when compared with formaldehyde. In the immobilized form, the accessibility of AOX to oxygen is limited and thus methanol oxidation is incomplete when compared with formaldehyde to generate formic acid, which is the reaction product detected by the H^+^ ion transducer. This shows that the formaldehyde biosensor based on microspheres with AOX immobilized is selective towards formaldehyde, especially above 3 mM of formaldehyde.

**Figure 7.**
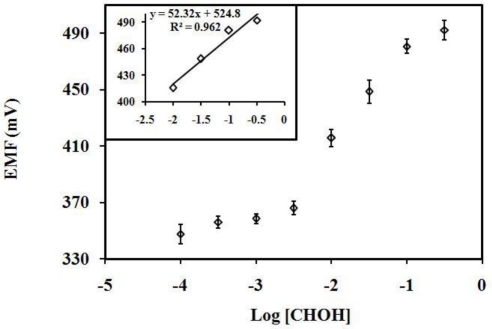
The response of a formaldehyde biosensor fabricated from poly(HEMA) film as an enzyme immobilization matrix towards various concentrations of formaldehyde in 10 mM Tris-HCl buffer at pH 6.5.

The improvement of the biosensor response that has been brought about by using poly(nBA-NAS) microspheres as the sensing matrix can be seen when compared with a formaldehyde biosensor where a poly(HEMA) film is used as an enzyme immobilization matrix. The response curve of a formaldehyde biosensor fabricated from a poly(HEMA) film with AOX entrapped is shown in Figure 7. The average biosensor response to formaldehyde was 54.91 ± 5.07 mV/decade in the linear range of 10.0–316.2 mM (R^2^ = 0.9684). Clearly, the biosensor without the use of microspheres as enzyme immobilization matrix demonstrated inferior linear response range and also poorer sensitivity.

Based on the comparison between biosensors from microspheres and film (Table 5), clearly there is a large improvement of the biosensor performance from the use of microspheres, namely the response time, detection limit and linear response range.

The detection limit of the biosensor with microsphere is lowered by eight times whilst the response time is also reduced by up to 10 times. Furthermore, biosensor based on AOX-poly(nBA-NAS) microspheres yielded better repeatability and reproducibility in sensitivity slopes less than 5% relative standard deviation (RSD) when compared with biosensor based on poly(HEMA) film where the RSD values are several times higher. Such improvement is probably attributable to the good diffusion properties provided by the surface reaction of AOX and formaldehyde and also the large surface area for reaction and enzyme binding sites from the use of microspheres compared with a thick film.

**Figure 8.**
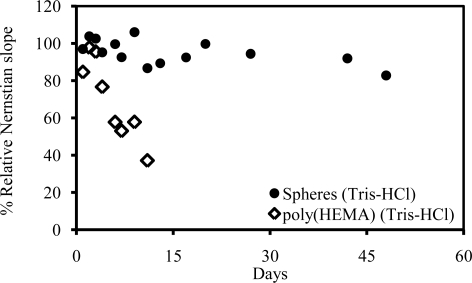
Comparison of the long term stability of biosensors based on AOX-poly(nBA-NAS) microspheres and poly(HEMA) film.

For continuous operation, the AOX-poly(nBA-NAS) microspheres-based biosensor could be used for up to 6 hours with analysis of approximately 104 samples where the response time for each sample is less than 8 s. Long term stability (Figure 8) of the biosensor is up to 48 days, with 80% of its initial response still achievable. On the contrary, the response of poly(HEMA) film based biosensor decrease to 43–58% of its initial value after a week of storage. In the poly(HEMA) film, the enzyme is only entrapped and this leads to leaching from the polymer matrix, thus results in shorter life span [45]. The improvement in the long and short term stability of the AOX-poly(nBA-NAS) microspheres based biosensor is due to the beneficial effect of covalent immobilization enzyme on the microsphere surface.

#### 3.3.4. Recovery Performance of Formaldehyde Biosensor and Comparison with Nash Method

The application of the AOX-poly(nBA-NAS) microspheres based formaldehyde biosensor was applied to analyze shrimp samples. The recovery of formaldehyde from the samples is shown in Table 6.

The percentages of recovery of formaldehyde from concentrations 2.19–3.7 mg kg^−1^ formaldehyde are between 91.5% and 105.1%, which showed that the biosensor is capable of determining formaldehyde in a real sample matrix. To verify this further, the Nash method, a standard method for formaldehyde analysis, was used for comparison with the biosensor method (Table 7). It was found that the formaldehyde concentration determined by both Nash and biosensor methods do not differ significantly (p > 0.05).

In terms of sensitivity, linear response range and response time, the SPE formaldehyde biosensor based on microspheres demonstrated improved performance when compared with potentiometric SPE biosensors from studies using AOX immobilized in poly(HEMA)-sol gel films [45] and ISFET based biosensors using formaldehyde dehydrogenase (FDH), AOX or yeast cell by Vianello *et al.* [56] and Korpan *et al.* [3]. The detailed comparison is summarized in Table 8. It is particularly interesting to note that biosensor of this work using microspheres showed much faster response time with a larger linear response range. This is probably attributable to the surface immobilization of AOX where diffusion of analyte and reaction products for detection is much improved compared with formaldehyde biosensors based on immobilization in thick films.

The conductometric biosensor [55] reported has linear response ranges varying from 0.05 to 500 mM and this is very much dependent on the buffer concentration used and the duration of AOX immobilization. For biosensor based on microspheres, the linear response range although narrower, it is less affected by the conditions of operation of the biosensor. Moreover, the formaldehyde biosensor using microspheres as reported here has several advantages over other electrochemical biosensors, e.g., better sensitivity compared with sensors based on coulometry [4] and shorter response time than conductometry based sensors [55]. Unlike amperometry based sensors [1, 57–63], for the biosensor reported here, addition of mediator is not necessary and it is less affected by oxygen concentration in the sample solution.

## 4. Conclusions

This work has shown that the use of hydrophobic acrylic microspheres for immobilization of enzyme, e.g., AOX can lead to an overall improvement in the analytical performance of the resulting biosensor. Considerable improvements are seen in the detection limit, response time, linear response range, reproducibility and repeatability. The successful covalent immobilization of the enzyme on the large surface area provided by the microspheres contributed to such improvements. Using acrylic microspheres, diffusion of reaction products was improved and this resulted in a large improvement in the response time of the biosensor. The formaldehyde biosensor based on acrylic microspheres showed good performance in the analysis of formaldehyde in food sample such as shrimp.

## Figures and Tables

**Table 1.** AOX-poly(nBA-NAS) microspheres based biosensor responses towards different concentrations of formaldehyde in Tris-HCl buffer pH 6.5.

**Buffer concentration (mM)**	**Sensitivity (mV/decade)**	**R^2^**	**Dynamic linear range (mM)**
0.1	52.55±6.43	0.9955	3.2–316.2
1.0	58.47±7.53	0.9719	1.0–100.0
10.0	60.17±6.00	0.9861	1.0–316.2

**Table 2.** A comparison of responses between sensors with and without AOX enzyme in various media.

**Media for preparing formaldehyde**	**Sensor without AOX**		**Formaldehyde biosensor with AOX**	

	**Sensitivity (mV/decade)**	**Linear range (mM)**	**Sensitivity (mV/decade)**	**Linear range (mM)**
Deionized water	37.52 ± 0.66	3.2–316.2	48.80 ± 1.95	1.0–316.2
0.1 mM Tris-HCl pH 6.5	41.91 ± 1.33	3.2–316.2	53.03 ± 1.13	1.0–316.2
10 mM Tris-HCl pH 6.5	43.50 ± 2.19	3.2–316.2	59.54 ± 0.65	1.0–316.2

**Table 3.** The effect of pH of the buffer on the response of the formaldehyde biosensor (n = 3, 10 mM Tris-HCl buffer).

**pH**	**Sensitivity (mV/decade)**	**Linear range (mM)**	**R^2^**
5.8	58.36 ± 3.15	1.0–100.0	0.9744
6.0	59.11 ± 3.00	1.0–100.0	0.9857
6.5	59.41 ± 0.66	0.3–316.2	0.9776
7.0	71.36 ± 2.29	1.0–316.2	0.9563
7.5	69.56 ± 15.00	10.0–316.2	0.9851

**Table 4.** The effect of total amount of poly(nBA-NAS) microspheres with immobilized AOX towards the formaldehyde biosensor response.

**Weight of spheres (mg)**	**Sensitivity (mV/decade)**	**Linear range (mM)**	**R^2^**
0.1	52.47 ± 1.24	0.3–316.2	0.9947
0.3	56.27 ± 0.99	0.3–316.2	0.9957
0.5	57.54 ± 2.23	0.3–316.2	0.9905
0.6	61.24 ± 3.34	0.3–316.2	0.9818

**Table 5.** Comparison of analytical performance between formaldehyde biosensors based on AOX-poly(nBA-NAS) and AOX-poly(HEMA) film (10 mM Tris-HCl buffer pH 6.5).

**Parameters**	**AOX-poly(nBA-NAS) microspheres**	**AOX-poly(HEMA) Film**
Sensitivity (mV/decade)	59.41 ± 0.66	54.91 ± 5.07
R^2^	0.9776	0.9684
Dynamic linear range (mM)	0.3–316.2	10.0–316.2
Detection limit (mM)	0.3	4.0
Response time (s)	1–8	10–85
Repeatability (RSD %)	1.11	9.23
Reproducibility (RSD %)	3.16	7.63

**Table 6.** Recovery performance of AOX-poly(nBA-NAS) microspheres based biosensors in the analysis of formaldehyde in shrimp samples.

**Spiked HCHO concentration (mg kg^−1^)**	**Determined concentration (mg kg^−1^) (n = 3)**	**Recovery (%)**
2.19	2.00 ± 0.09	91.5
2.71	2.65 ± 0.08	97.8
3.16	3.25 ± 0.09	102.6
3.56	3.58 ± 0.03	100.5
3.74	3.93 ± 0.18	105.1

**Table 7.** Comparison between AOX-poly(nBA-NAS) microspheres based biosensor and Nash method in the determination of formaldehyde in shrimp sample.

**Nash Standard method (mg kg^−1^) (n = 3)**	**Biosensor method (mg kg^−1^) (n = 3)**
17.80 ± 1.65	17.47 ± 2.02
36.21 ± 0.50	36.12 ± 1.12
59.99 ± 0.14	59.53 ± 1.44
78.76 ± 1.02	75.68 ± 1.43
87.67 ± 1.73	87.58 ± 1.43

**Table 8.** Comparison between AOX-poly(nBA-NAS) microspheres based biosensor and reported potentiometric formaldehyde biosensors.

**Parameters**	**This study**	**Siti*et al.*[45]**		**Korpan*et al.*[3]**		**Vianello*et al.*[56]**
Enzyme	AOX	AOX	AOX^α^	AOX^α,a^	AOX^α,b^	FDH
Sensitity (mV/decade)	59.4 ± 0.7	43.9 ± 2.1	26.0	18.0	—	24.5
Dynamic linear range (mM)	0.3–316.2	1.0–100.0	5.0–200.0	5.0–200.0	5.0–50.0	0.01–0.2
Detection limit (mM)	0.3	—	—	—	—	0.01
Response time (s)	1–8	—	10–60	10–60	60–120	67
Reproducibility (RSD %)	3	15	2	2	5	—
Long term stability (days)	48	—	60	90	30	—

α Korpan *et al.* [3];

a Partially purified AOX from catalase deficient mutant *Hansenula polymorpha*;

b Permeabilised cells as a source of AOX from whole yeast cells *Hansenula polymorpha.*
